# Vanadium-Doped Bioactive Glass-Modified GelMA/CMCS/HA Injectable Hydrogel for Osteosarcoma Postoperative Therapy and Bone Regeneration

**DOI:** 10.3390/ma19102086

**Published:** 2026-05-15

**Authors:** Dazhong Jin, Miaomiao He, Guangfu Yin

**Affiliations:** College of Biomedical Engineering, Sichuan University, Chengdu 610065, China; jx1429476207@163.com (D.J.); hemmiao@scu.edu.cn (M.H.)

**Keywords:** osteosarcoma, gelatin methacryloyl, chitosan, hyaluronic acid, vanadium

## Abstract

Surgical intervention is a primary treatment for osteosarcoma, often resulting in a tumorous bone defect with an irregular shape. Postoperative management is essential to minimize tumor recurrence risks and promote bone regeneration. To address these issues, we developed a multifunctional injectable, rapidly photo-curable hydrogel composed of gelatin methacryloyl/carboxymethyl chitosan/hyaluronic acid (GelMA/CMCS/HA), modified with vanadium-doped mesoporous bioactive glass (VMBG). The exceptional injectability enables seamless adaptation to irregular bone defects, offering a significant advantage over preformed implants, while the rapid photocurability of the hydrogel ensures stable fixation within minutes, thereby reducing potential risks during surgery. Furthermore, this platform exhibits dual therapeutic efficacy, characterized by antitumor activity and osteogenic induction. In vitro assessments demonstrated that V(V)/V(IV) valence cycling-driven ROS generation mediated its potent antitumor efficacy. Additionally, concurrent enhancement of alkaline phosphatase activity and osteogenic marker expression validated its osteogenic potential. The CMCS incorporation promoted healing at the defect site, while the HA addition created binding sites for cell adhesion and growth, thereby improving scaffold bioactivity. Collectively, this study presents the development and validation of a multifunctional GelMA/CMCS/HA hydrogel, highlighting its dual capability for bone regeneration and tumor suppression within tumor-associated bone microenvironments.

## 1. Introduction

Osteosarcoma is the most common primary malignant bone tumor, predominantly affecting adolescents and young adults aged 10–25 years, with a secondary incidence peak in adults over 60 years [1,2,3]. Approximately 60% of cases occur in patients under 25 years of age. Studies have implicated many inducing factors, including genetic predisposition, ionizing radiation exposure, viral infection, and chemical exposure [4,5]. Currently, surgical intervention remains the mainstay of treatment. However, persistent challenges, including metastatic recurrence, incomplete tumor resection [6,7,8], and postoperative delayed bone healing, necessitate urgent resolution. Therefore, the development of injectable biomaterials capable of effectively suppressing residual tumor cells while promoting osteogenesis represents an urgent clinical need, offering significant potential for enhancing bone regeneration in osteosarcoma resection patients.

Hydrogels are a class of highly hydrophilic three-dimensional network structures characterized by high water content and excellent biocompatibility [9]. Their unique physicochemical properties make them ideal candidates for mimicking the cellular microenvironment, leading to widespread applications in the biomedical field [10,11,12,13]. Carboxymethyl chitosan (CMCS) is an amphoteric polyelectrolyte derived from the carboxymethylation of chitosan, demonstrating superior water solubility, antibacterial activity, wound-healing properties, and biocompatibility [14,15]. Hyaluronic acid (HA) is a linear anionic polysaccharide naturally present in human connective tissues, characterized by unique rheological properties and excellent biocompatibility. It promotes cell proliferation and migration and regulates the differentiation of BMSCs, thereby accelerating bone defect healing [16,17]. Injectable hydrogels and 4D materials hold great promise for the treatment of irregular wound defects, as they can conform to the shape of the defect without leaving voids [18,19,20,21]. Gelatin methacryloyl (GelMA) is a photosensitive biomaterial [22]. In 2000, Van Den Bulcke and colleagues first reported the modification of gelatin with methacrylic anhydride (MA), successfully introducing photosensitive carbon–carbon double bonds onto the gelatin backbone, enabling free radical polymerization under ultraviolet or blue light irradiation, thus forming a three-dimensional cross-linked network [23]. This pioneering work addressed the inherent limitations of native gelatin, namely poor stability and dissolution tendency under physiological conditions, while preserving its excellent bioactivity and endowing it with photosensitivity. Decades of research have well established the mechanisms governing the tunable properties of GelMA. However, certain challenges remain, such as its relatively poor mechanical performance in bone tissue engineering scaffolds, which limits its application in bone defect repair.

In recent years, bioactive hydrogels incorporating inorganic components have demonstrated superior biocompatibility and osteogenic potential, providing novel therapeutic strategies for bone defect regeneration [24,25]. Mesoporous bioactive glass (MBG) is an advanced biomaterial class that actively promotes host tissue regeneration through direct chemical bonding with surrounding tissues. MBG features a well-defined mesoporous structure, high porosity, and unique biological properties [26,27]. Moreover, MBG also serves as an efficient carrier for therapeutic metal ions, enabling sustained ion release. Vanadium (V) is a transition metal with variable valence states and is considered an essential trace element in the human body, playing an important role in regulating metabolic activities [28,29]. Previous studies have shown that V improves glucose and lipid metabolism, promotes insulin signaling transduction, and exerts hypoglycemic and insulin-mimetic effects. Notably, distinct V valence states drive divergent biological functions. Pentavalent V (V(V)) regulates the early and mid-stage osteogenic differentiation of bone marrow mesenchymal stem cells (BMSCs), while tetravalent V (V(IV)) exerts antitumor effects through multiple mechanisms and regulates late-stage osteogenic differentiation as well as angiogenesis [30,31,32].

In this study, we propose a multifunctional injectable double-network hydrogel, composed of GelMA/CMCS/HA (abbreviated as GCH), designed to simultaneously facilitate bone defect repair and eliminate residual tumor cells after osteosarcoma resection. The primary network of this hydrogel is formed through hydrogen bonding, while the secondary network is crosslinked in situ upon exposure to blue light (λ = 405 nm). This hydrogel can be injected into the defect site via a syringe and subsequently stabilized by photo-crosslinking under blue light irradiation to perfectly conform to the defect geometry. CMCS contributes to antibacterial activity at wound sites while synergistically enhancing cell proliferation with HA. To confer multifunctionality, vanadium species (V) were doped in MBG to form VMBG, which was incorporated into the hydrogel matrix, ultimately yielding the GCH/VMBG composite hydrogel. We postulate that the sustained release of tetravalent vanadium species from the hydrogel exerts effective antitumor activity during the initial post-implantation phase. Subsequently, the released pentavalent and tetravalent vanadium species collectively regulate the osteogenic differentiation of BMSCs across early, middle, and late stages, concurrently suppressing tumor recurrence and promoting bone regeneration. Meanwhile, the gradually degraded CMCS and HA provide binding sites and space, synergistically facilitating cell adhesion, proliferation, and new bone tissue formation. To validate this hypothesis, we evaluated the physicochemical properties of this hydrogel under physiological conditions, including mechanical strength, swelling behavior, degradation profile, and ion-release behaviors. Subsequently, we evaluated the in vitro biocompatibility of this hydrogel and validated its antitumor efficacy against osteosarcoma cells and osteogenic promotive effects. Through comprehensive material characterization and in vitro validation, this composite hydrogel system exhibited significant dual functionality: promoting bone tissue regeneration while suppressing tumor progression, demonstrating therapeutic potential for tumor-associated bone defects.

## 2. Materials and Methods

### 2.1. Materials and Reagents

GelMA was purchased from Engineering For Life (EFL, Suzhou, China). CMCS, cetyltrimethylammonium bromide (CTAB), lithium phenyl (2,4,6-trimethylbenzoyl) phosphinate (LAP), ammonium hydroxide (GR, 25–28%), triethyl phosphate (TEP), and ascorbic acid (ASA) were obtained from Aladdin (Shanghai, China). Sodium hyaluronate (40–100 kDa) and sodium orthovanadate were purchased from Macklin (Shanghai, China). Tetraethyl orthosilicate (TEOS) and calcium nitrate tetrahydrate were purchased from Kelong (Chengdu, China). Ethyl acetate (EA) was obtained from Jinshan (Chengdu, China). Phosphate-buffered saline (PBS, 1×) and simulated body fluid (SBF) were purchased from Labgic (Beijing, China).

Related biological reagents were purchased from Cyagen (Suzhou, China), Procell (Wuhan, China), and ZETA Life (Xi’an, China). L929, UMR-106 and BMSC were obtained from Immocell (Xiamen, China). Assay kits were purchased from Beyotime (Shanghai, China).

### 2.2. Synthesis of Bioactive Glasses

MBG and VMBG were synthesized using a micro-sacrificial template method [33]. Briefly, 0.7 g of CTAB was dissolved in 33 mL of ultrapure water (UPW) under stirring for 15 min, followed by the addition of 10 mL of ethyl acetate with stirring for 30 min, and then 7 mL of ammonium hydroxide (1 mol/L) with stirring for an additional 15 min. Subsequently, the raw materials were added according to the designated ratios. Specifically, 3.6 mL of TEOS was added and stirred for 30 min, followed by 0.36 mL of TEP with stirring for 30 min. Finally, 2.28 g of Ca(NO_3_)_2_·4H_2_O, 51.69 mg of Na_3_VO_4_, and 6.19 mg of ASA were added sequentially, and the mixture was stirred until complete dissolution. The reaction was allowed to proceed under continuous stirring in a 40 °C water bath for 5 h. The resulting precipitate was collected by centrifugation and washed alternately with anhydrous ethanol and ultrapure water three times. The product was then dried in an oven at 60 °C for 12 h, yielding a white agglomerated solid. The dried product was calcined in a programmable muffle furnace at 700 °C for 5 h with a heating rate of 2 °C/min to remove the template and facilitate structural reconstruction of the crystal framework. After calcination, the product was ground to obtain the VMBG.

MBG was composed of SiO_2_, CaO, and P_2_O_5_, and the molar ratios of constituent elements were as follows: Si (57.2%), Ca (35.3%), and P (7.5%). For VMBG, V_2_O_5_ was additionally introduced into the composition, with the V loading of 1%, while the molar ratios of Si, Ca, and P remained unchanged.

### 2.3. Characterization of Bioactive Glasses

The morphologies of MBG and VMBG were characterized using scanning electron microscopy (SEM) and transmission electron microscopy (TEM). Fourier-transform infrared spectroscopy (FTIR) was employed for further structural analysis. The pore size and specific surface area were determined using the Brunauer–Emmett–Teller (BET) and Barrett–Joyner–Halenda (BJH) methods. Crystal phase transitions and mineralization processes were analyzed using X-ray powder diffraction (XRD). Elemental distribution, composition, and valence states of VMBG were investigated using energy-dispersive X-ray spectroscopy (EDS) and X-ray photoelectron spectroscopy (XPS).

In vitro mineralization assays were performed using simulated body fluid (SBF). MBG and VMBG were immersed in SBF solution and placed in an orbital shaker set at 150 rpm and 37 °C for 14 days. The mineralized samples were collected by centrifugation and drying.

### 2.4. Preparation of Hydrogels

#### 2.4.1. Preparation of LAP Photoinitiator Stock Solution

0.05 g of the photoinitiator was dissolved in 20 mL of PBS to obtain a 0.25% (*w*/*v*) stock solution, which was stored in a light-protected brown bottle at 4 °C.

#### 2.4.2. Synthesis of Hydrogels

The bone-filling hydrogels were composed of GelMA, CMCS, and HA. First, GelMA, CMCS, and HA were dissolved in the LAP stock solution at 40 °C in the dark, achieving final concentrations of 10%, 2%, and 1% (*w*/*v*), respectively. Subsequently, VMBG was added to the pre-hydrogel solution and dispersed using ultrasonication. Finally, the mixture was irradiated with blue light (λ = 405 nm) for 2 min to allow photo-crosslinking, resulting in the formation of a dual-network hydrogel. Hydrogels are sterilized by UV irradiation for half an hour before in vitro experiments. The hydrogel without MBG was designated as GCH, the hydrogel containing MBG was designated as GCH/MBG, and the hydrogel containing VMBG was designated as GCH/VMBG.

### 2.5. Characterization of Hydrogels

GCH, GCH/MBG, and GCH/VMBG hydrogels were characterized by SEM, FTIR, rheological property measurements, and compressive strength testing, as well as swelling and degradation behavior analyses.

Ion-release tests were conducted by immersing GCH/VMBG hydrogel in SBF at a VMBG concentration of 1 mg/mL. Supernatants were collected at various time points, and the concentrations of silicon, calcium, phosphorus, and vanadium in each sample were assessed using inductively coupled plasma mass spectrometry (ICP-MS).

### 2.6. Biocompatibility

Cell viability was assessed to evaluate the biocompatibility of the hydrogels. The Cell Counting Kit-8 (CCK-8) assay was performed to determine the in vitro cytotoxicity of L929 cells and BMSCs cultured in 96-well plates following exposure to various concentrations of hydrogel extracts. After the addition of CCK-8 solution and incubation at 37 °C for 2 h, the absorbance at 450 nm was measured to evaluate cell viability. In addition, live/dead cell staining was performed using a Calcein/PI staining kit (Beyotime, Shanghai, China), and fluorescence imaging was conducted after cell culture.

### 2.7. In Vitro Antitumor Assay

The CCK-8 assay was performed to evaluate the in vitro cytotoxicity of different hydrogel extracts on UMR-106 cells cultured in 96-well plates. After the addition of CCK-8 solution and incubation at 37 °C for 2 h, the absorbance at 450 nm was measured to assess cell viability. Additionally, live/dead cell staining was conducted using a Calcein/PI staining kit (Beyotime, Shanghai, China), and fluorescence imaging was performed following cell culture.

To analyze the generation of reactive oxygen species, ROS staining was performed using a ROS assay kit (CA1420, Solarbio, Beijing, China), followed by fluorescence imaging after cell culture.

### 2.8. In Vitro Osteogenesis Assay

#### 2.8.1. Cell Culture

Third-passage BMSCs were used for all experiments. To evaluate the potential of hydrogels to promote osteogenic differentiation of BMSCs, hydrogels (0.1 g/mL) were immersed in culture medium for 72 h. Subsequently, cells were cultured in different hydrogel extracts for 7 and 14 days. The culture medium was refreshed every three days for each group.

#### 2.8.2. Alkaline Phosphatase and Alizarin Red S Staining

BMSCs were treated with different hydrogel extracts (control, GCH, GCH/MBG, and GCH/VMBG) at a concentration of 0.1 g/mL for 7 and 14 days. The cells were washed with PBS and fixed with 4% paraformaldehyde solution. Alkaline phosphatase (ALP, Solarbio, Beijing, China, G1480) staining was performed using an ALP staining kit and observed under a microscope. Quantitative analysis was conducted using an alkaline phosphatase assay kit, with absorbance measured at 405 nm. After 14 days of induction, cells were washed with PBS, fixed with 4% paraformaldehyde solution, and stained with Alizarin Red S (ARS, Solarbio, Beijing, China, G1450). Observations were made under a microscope.

#### 2.8.3. Osteogenic Protein Analysis

BMSCs were treated with different hydrogel extracts (control, GCH, GCH/MBG, and GCH/VMBG) for 3 and 6 days. Cell culture supernatants from each treatment group were collected by centrifugation, and the expression levels of BMP-2, Col-I, and OCN were analyzed using ELISA kits (Beyotime, Shanghai, China).

### 2.9. Statistical Analysis

Each data point represents at least three independent replicate experiments. All values are expressed as the mean ± standard deviation. Statistical significance was analyzed using two-way analysis of variance (ANOVA), with significance levels set as follows: * *p* < 0.05; ** *p* < 0.01; and *** *p* < 0.001.

## 3. Results and Discussion

### 3.1. Characteristics of Bioactive Glasses

#### 3.1.1. Morphology of MBG and VMBG

**Figure 1:**
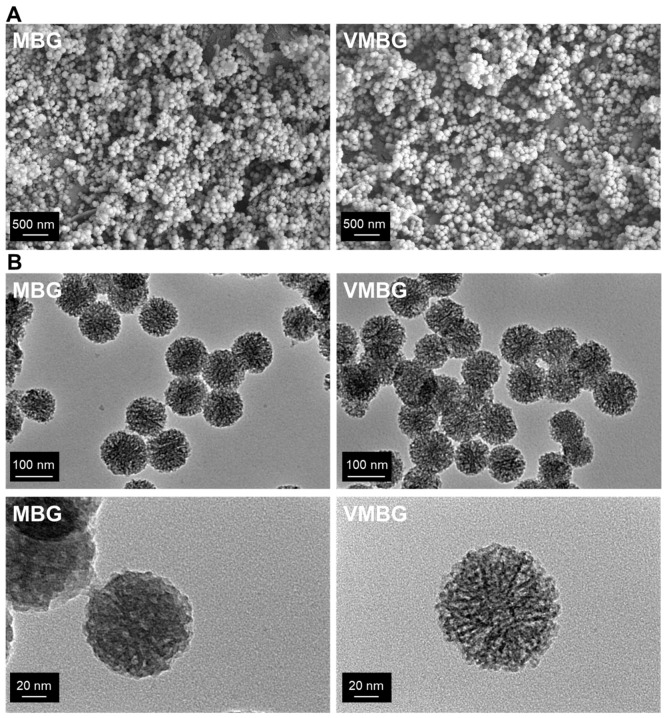
Morphological characteristics of MBG and VMBG: (**A**) SEM (secondary electron) and (**B**) TEM images at different magnifications.

Morphology and size of MBG and VMBG were characterized using SEM and TEM (Figure 1). Both MBG and VMBG exhibited uniform spherical morphology with rough surfaces and diameters of approximately 100 nm. The introduction of V did not significantly alter the size or morphology of MBG. SEM images revealed satisfactory dispersibility of MBG and VMBG particles. TEM images showed the presence of randomly oriented interconnected channels within the MBG and VMBG particles.

#### 3.1.2. Elemental Distribution of VMBG

**Figure 2:**
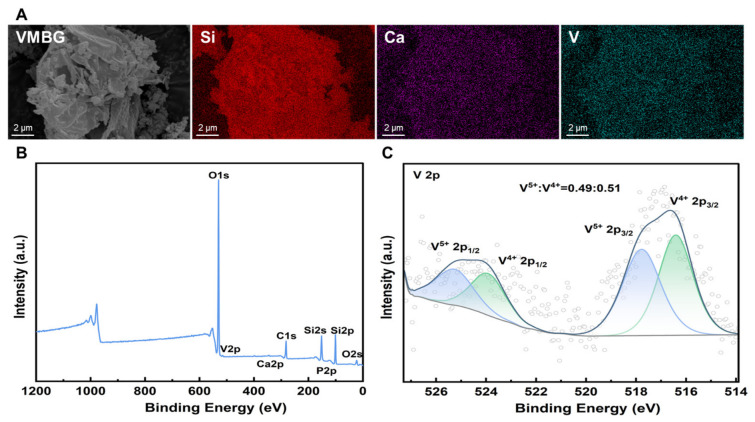
Elemental distribution and chemical states of VMBG: (**A**) elemental analysis of O, Si, Ca, and V in VMBG; (**B**) XPS survey spectrum; (**C**) XPS spectrum of V 2p.

Elemental distribution and valence states of VMBG were characterized using EDS and XPS. EDS mapping (Figure 2A) revealed that the four elements were uniformly distributed throughout the glass phase, with no observable aggregation of the doped V, indicating that vanadium was incorporated into the silica network of MBG in the form of ions or ionic clusters. XPS analysis (Figure 2B) showed the presence of V, Si, Ca, and P, further confirming the successful doping of V. High-resolution XPS spectra of the V 2p (Figure 2C) revealed that, upon the addition of ascorbic acid as a reducing agent, a portion of pentavalent V(V) was reduced to tetravalent V(IV), resulting in an approximate 1:1 ratio of the two valence states after reduction.

#### 3.1.3. Structure of MBG and VMBG

**Figure 3:**
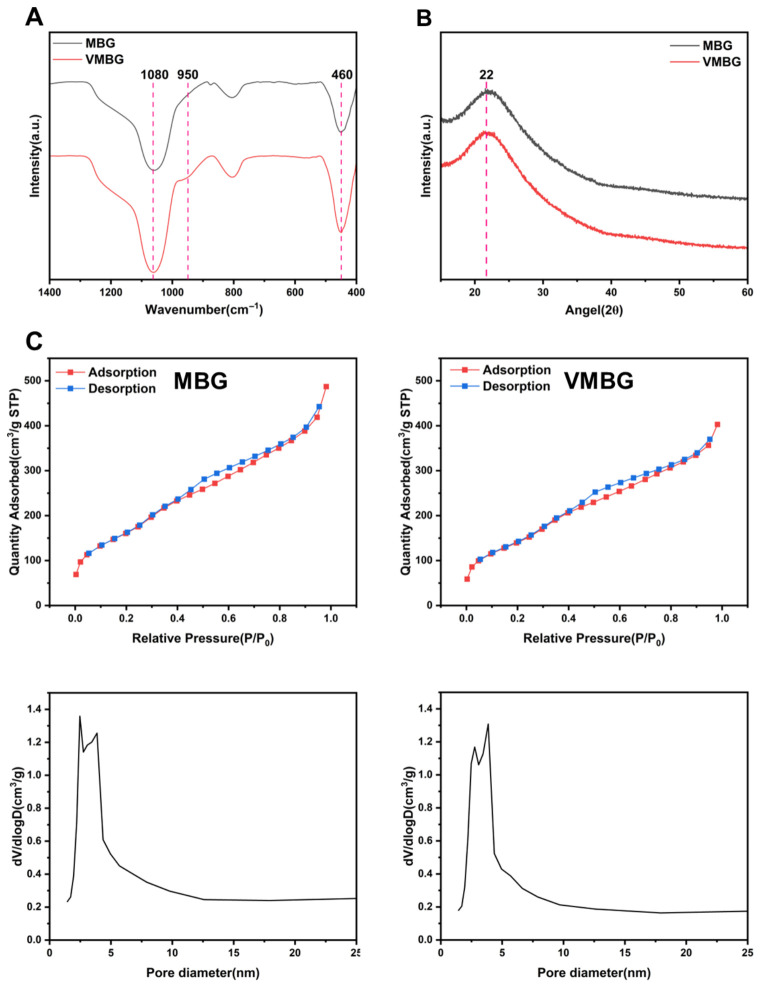
Structural characterization of MBG and VMBG: (**A**) FTIR spectra; (**B**) XRD patterns; (**C**) N_2_ adsorption–desorption isotherms and pore size distributions.

Structures of MBG and VMBG were characterized by XRD, FTIR, and N_2_ adsorption–desorption measurements. FTIR spectra (Figure 3A) revealed a peak at 1080 cm^−1^ corresponding to the asymmetric stretching vibration of Si-O-Si and a peak at 460 cm^−1^ attributed to the bending vibration of Si-O-Si. Additionally, upon the incorporation of trace amounts of V, a new peak at 950 cm^−1^ was observed. XRD patterns (Figure 3B) showed that both MBG and VMBG exhibited only a broad SiO_2_ peak centered at approximately 2θ = 22°, with no diffraction peaks corresponding to V compounds, indicating that doped V did not significantly alter the amorphous structure of MBG.

N_2_ adsorption–desorption isotherms of MBG and VMBG (Figure 3C) both exhibited type IV hysteresis loops. According to the International Union of Pure and Applied Chemistry (IUPAC) classification, it is characteristic of mesoporous materials. The Brunauer–Emmett–Teller (BET) specific surface area of MBG was detected to be 607.5 m^2^/g, while the Barrett–Joyner–Halenda (BJH) specific surface area (desorption) was 722.8 m^2^/g. The pore volume and average pore diameter of MBG were 0.755 cm^3^/g and 4.974 nm, respectively. For VMBG, the BET specific surface area was 527.2 m^2^/g, and the BJH specific surface area (desorption) was 630.8 m^2^/g, with a pore volume of 0.625 cm^3^/g and an average pore diameter of 4.741 nm. After V doping, a slight decrease in specific surface area, pore volume, and average pore diameter was observed, which may be attributed to the influence of V incorporation on the silica network.

#### 3.1.4. In Vitro Mineralization of MBG and VMBG

**Figure 4:**
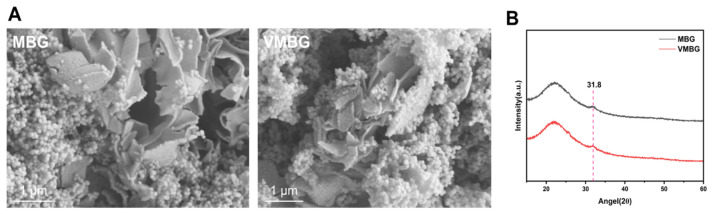
In vitro mineralization of MBG and VMBG soaked in SBF: (**A**) SEM images; (**B**) XRD patterns.

SEM images of MBG and VMBG after 14-day SBF immersion (Figure 4A) displayed distinct two-dimensional lamellar hydroxyapatite (HAP) formations. Corresponding XRD patterns (Figure 4B) revealed a new diffraction peak at approximately 2θ = 31.8° post-immersion, indexed to the (211) crystal plane of HAP. These findings collectively confirm SBF-induced HAP crystallization on both materials. Formation of HAP is beneficial for osseointegration, demonstrating the surface bioactivity of MBG.

### 3.2. Characteristics of Hydrogels

#### 3.2.1. Morphology of Hydrogels

**Figure 5:**
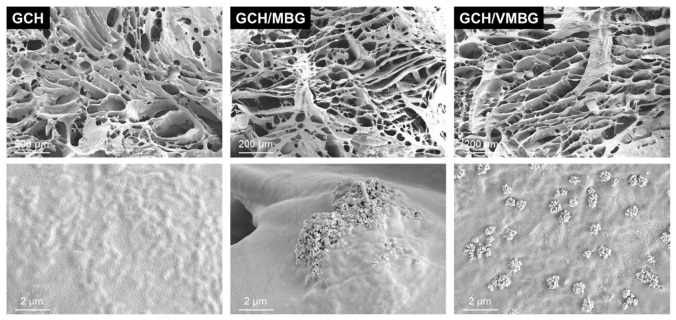
SEM images of GCH, GCH/MBG, and GCH/VMBG at different magnifications.

Injectable hydrogels possess the ability to conform to irregular defects, offering great potential as scaffolds for bone tissue engineering. Upon mixing GelMA, CMCS, and HA, hydrogen bonding between CMCS and HA formed the primary crosslinked network. At this stage, the hydrogel exhibited no fixed shape and demonstrated favorable flowability, allowing it to be injected into bone defects in any shape. Subsequently, upon irradiation under 405 nm blue light, the photoinitiator LAP underwent homolytic decomposition to generate free radicals, leading to the formation of the secondary network and imparting the hydrogel with stable and superior properties. SEM analysis (Figure 5) revealed a hierarchically porous network within the hydrogel, with widely distributed pore sizes of 20–200 μm. Macropores provided ample space for cell adhesion and growth, whereas micropores facilitated nutrient transport, thereby supporting the regeneration of defective tissues. At higher magnification (100×), MBG and VMBG bioactive glass particles were observed firmly anchored on the hydrogel scaffold, with localized agglomeration.

#### 3.2.2. Structure of Hydrogels

**Figure 6:**
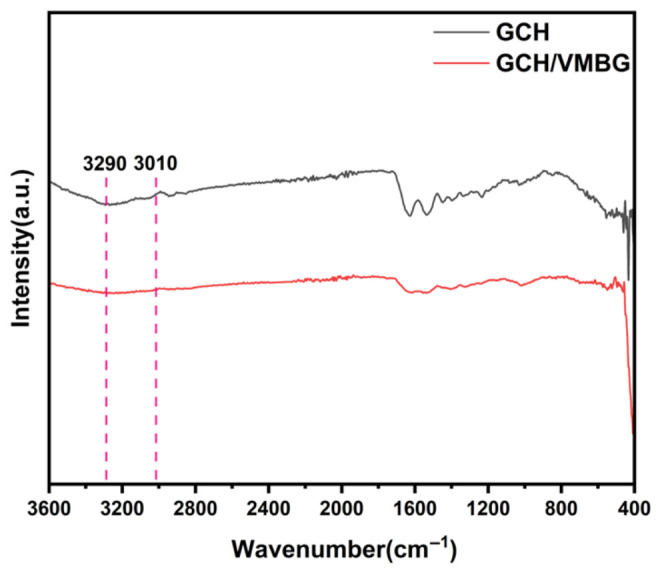
FTIR spectra of GCH and GCH/VMBG.

FTIR analysis of these hydrogels (Figure 6) showed that a broad peak near 3290 cm^−1^, assigned to O–H/N–H stretching vibrations mediated by hydrogen bonding, was weakened upon VMBG incorporation, indicating partial disruption of the hydrogen bonding network. Disappearance of the peak at 3010 cm^−1^ (characteristic of =C–H stretching in methacryloyl groups) confirmed the effective decomposition of C=C bonds in GelMA by LAP-generated free radicals, enabling 3D network formation via C–C crosslinking.

#### 3.2.3. Mechanical Properties of Hydrogels

**Figure 7:**
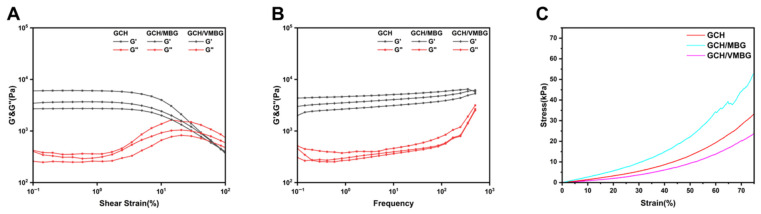
Mechanical properties of GCH, GCH/MBG, and GCH/VMBG: (**A**) G′ and G″ of hydrogels scanned under shear strain and (**B**) under frequency. (**C**) compressive strength curve of hydrogels.

The hydrogels displayed characteristics of a three-dimensional network structure. Strain sweep rheology (Figure 7A) revealed that the crossover points of the storage modulus (G′) and loss modulus (G″) shifted right upon bioactive glass incorporation, indicating enhanced critical strain tolerance. Frequency sweep rheology (Figure 7B) confirmed that all three hydrogels displayed typical gel behavior, characterized by G′ being significantly greater than G″ and G′ remaining largely insensitive to changes in frequency. Compressive strength testing (Figure 7C) showed that the hydrogels began to exhibit inelastic deformation at approximately 60% strain. The Young’s moduli of GCH, GCH/MBG, and GCH/VMBG were determined to be 35.8 kPa, 63.2 kPa, and 25.8 kPa, respectively. Osteoblasts and bone marrow mesenchymal stem cells (BMSCs) are the primary cells that perceive mechanical stimuli from the surrounding matrix and respond accordingly throughout bone regeneration and remodeling. It has been reported that GelMA hydrogels with a compressive strength in the range of 20–30 kPa significantly promote the osteogenic differentiation of BMSCs through stiffness-mediated activation of the FAK/paxillin signaling pathway [34].

#### 3.2.4. Swelling and Degradation of Hydrogels

**Figure 8:**
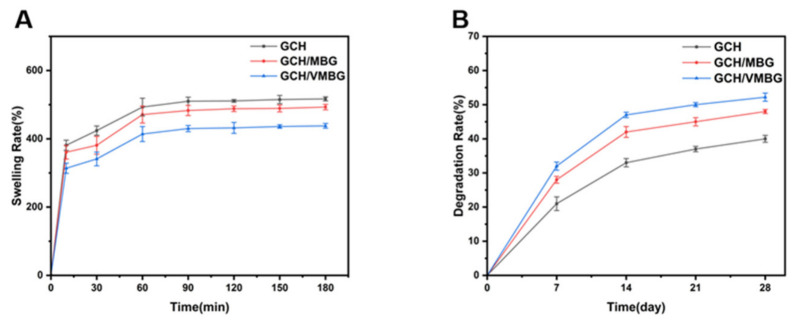
Swelling and degradation of GCH, GCH/MBG, and GCH/VMBG: (**A**) swelling rate; (**B**) degradation rate.

Excessive swelling alters the mechanical properties of hydrogels, thereby affecting the mechanical environment and potentially leading to adverse bone remodeling [35]. All hydrogels reached swelling equilibrium within 60 min (Figure 8A), ensuring rapid absorption of tissue fluid in defect sites after implantation and creating an environment conducive to cell growth. The controlled swelling ratio (400–500%) preserved the hydrogel’s structural integrity, maintaining a stable mechanical microenvironment conducive to osteoblast activity and mineralization. Degradation kinetics (Figure 8B) showed an initial rapid phase, followed by a progressive slowing due to polymer network stabilization. The incorporation of bioactive glass accelerated the degradation process. First, the release of Ca^2+^ ions from MBG and VMBG facilitates ion exchange with H^+^ ions in the solution, leading to the formation of a silica-rich gel layer on the surface of the bioactive glass. This process disrupts the interaction between MBG/VMBG and the hydrogel network, further destabilizing the network structure and promoting degradation. Additionally, the VMBG incorporation and subsequent degradation within hydrogels reduce network uniformity and increase the number of pores, thereby increasing the specific surface area of the hydrogels and accelerating the degradation process. Upon implantation, the hydrogel initially swells in a controlled manner and subsequently degrades, facilitating the repair of the defect site.

#### 3.2.5. Ion Release from Hydrogels

**Figure 9:**
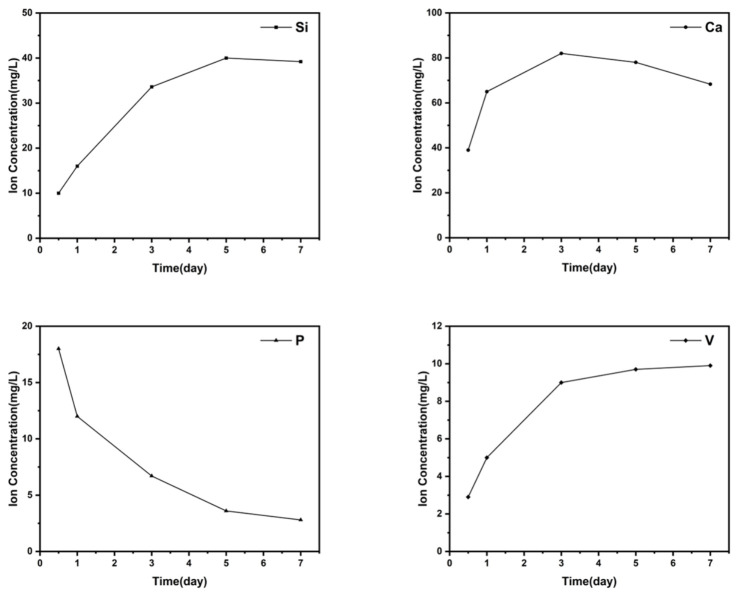
Release of Si, Ca, P, and V from hydrogel soaked in SBF.

The release of ions is a crucial way for the hydrogel to exert its biological functions. Release profiles of Si, Ca, P, and V from the hydrogel (Figure 9) displayed a sustained release pattern. Si and Ca concentrations in soaking media exhibited an initial increase followed by a decrease, whereas P concentration in media continuously decreased. This observation may be attributed to the relatively low P content in the bioactive glass. The P was likely released completely into the SBF during the early stage, triggering gradual HAP formation with concomitant P consumption. V concentration in media increased slowly during the initial phase (within 72 h) and then reached a plateau.

### 3.3. In Vitro Cytotoxicity of Hydrogels

**Figure 10:**
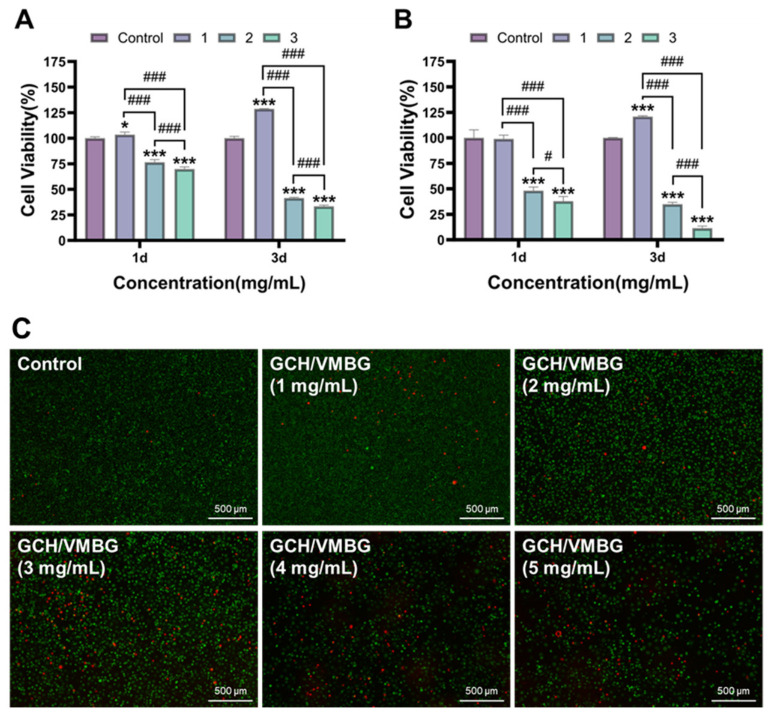
Cytotoxicity of hydrogels with different VMBG contents. (**A**) cell viability of L929; (**B**) cell viability of BMSC; (**C**) live/dead staining of L929 for 3 days. (* *p* < 0.05, *** *p* < 0.001; # *p* < 0.05; ### *p* < 0.001).

The cytotoxicity of hydrogels at varying VMBG concentrations (1, 2, and 3 mg/mL) was evaluated using the CCK-8 assay and live/dead staining [36]. When the content of VMBG exceeded 1 mg/mL, cell viability decreased to below 80%. The experimental results indicated that a VMBG concentration of 1 mg/mL exhibited optimal biocompatibility and proliferative effects (Figure 10). Notably, after 3 days of co-culturing, the group with a VMBG concentration of 1 mg/mL showed statistically significant differences compared with the control group, with cell viabilities of L929 and BMSCs reaching 128.6 and 120.9% (*p* < 0.001), respectively. In subsequent experiments, the concentrations of MBG and VMBG were set to 1 mg/mL.

### 3.4. Antitumor Activity of Hydrogels

**Figure 11:**
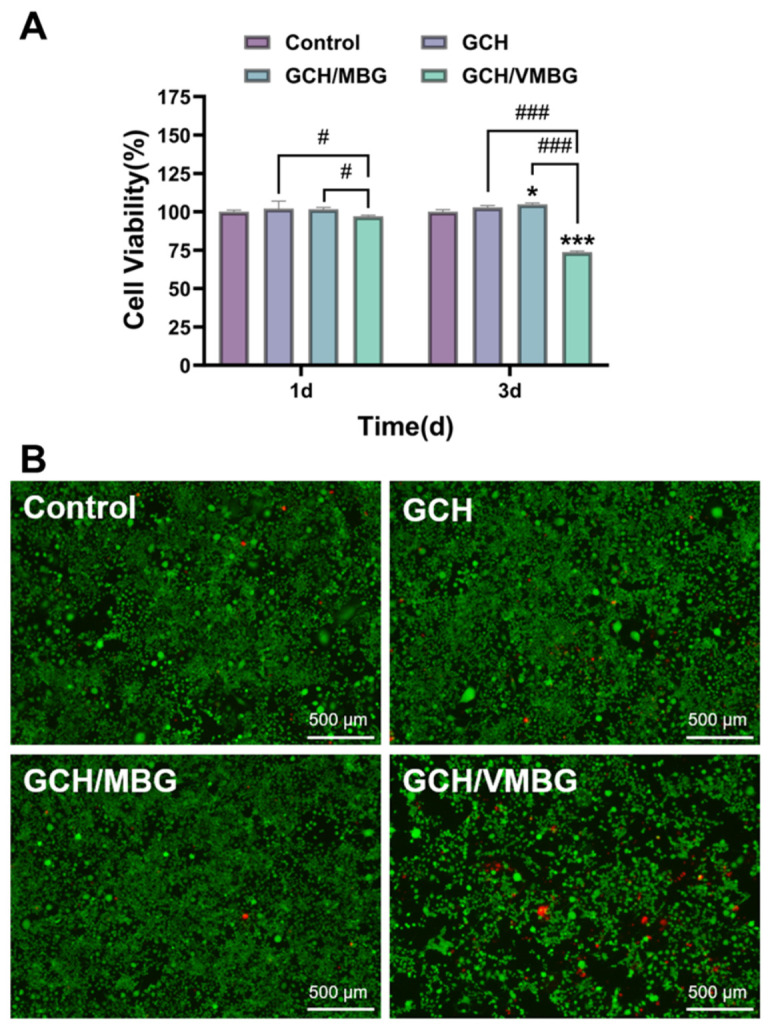
Antitumor effects of hydrogels: (**A**) cell viability and (**B**) live/dead staining of UMR-106 cultured for 3 days in media containing different hydrogel extracts. (* *p* < 0.05, *** *p* < 0.001; # *p* < 0.05, ### *p* < 0.001).

Hydrogels with antitumor properties can effectively eliminate residual tumor cells at bone defect sites, thereby preventing recurrence. Their antitumor effects on osteosarcoma tumor cells (UMR-106 line) were evaluated via CCK-8 assay and live/dead staining. CCK-8 results (Figure 11) showed that both GCH and GCH/MBG exhibited no significant cytotoxicity toward UMR-106 (*p* > 0.05), while GCH/VMBG demonstrated cytotoxicity against tumor cells, with cell viability reduced to 73.6% (*p* < 0.001).

The chemodynamical therapy based on Fenton or Fenton-like reactions has attracted increasing attention. It utilizes excess H_2_O_2_ to generate highly oxidizing hydroxyl radicals (·OH) in acidic conditions, inducing tumor cell death through oxidative damage to DNA, lipids, and proteins [37]. In tumor microenvironments, tetravalent V(IV) catalyzes a Fenton-like reaction to generate hydroxyl radicals (·OH), while the resultant pentavalent V(V) is reduced back to V(IV) by glutathione (GSH):

V(IV) + H_2_O_2_ → V(V) + ·OH + OH^−^

V(V) + GSH → V(IV) + GSSG + H^+^

**Figure 12:**
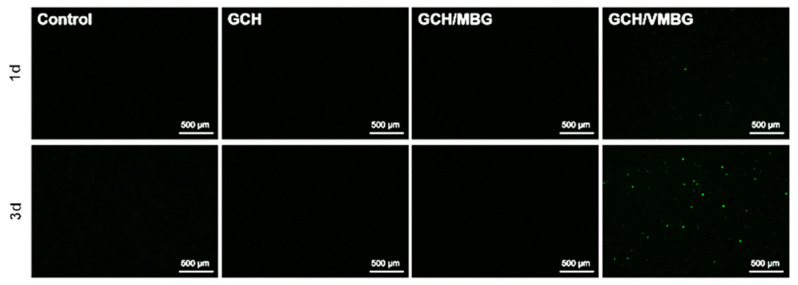
ROS generation in UMR-106 treated with different hydrogels.

Thus, the mixed-valence V(IV/V)-doped MBG sustainedly catalyzes ROS generation through valence cycling, thereby inducing tumor cell apoptosis. A ROS assay kit was used to evaluate ROS generation catalyzed by hydrogels. ROS production was observed only in the GCH/VMBG group, and the amount of ROS gradually increased over time (Figure 12). Notably, ROS are the primary source of the antitumor activity of VMBG, and this activity is positively correlated with the amount of generated ROS.

### 3.5. In Vitro Osteogenic Promotion

**Figure 13:**
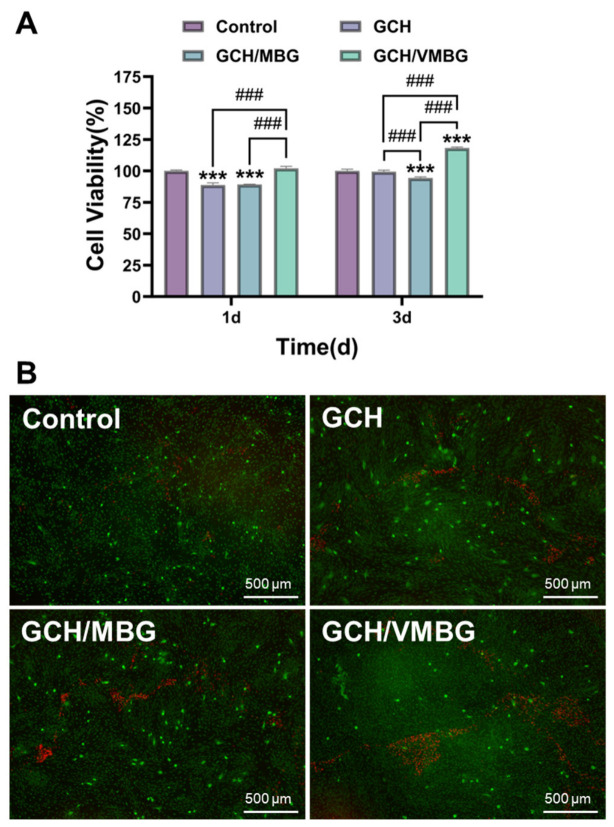
Effects of hydrogels on BMSC proliferation: (**A**) cell viability and (**B**) live/dead staining of BMSCs cultured for 3 days in media containing different hydrogel extracts. (*** *p* < 0.001; ### *p* < 0.001).

The effects of the hydrogels on osteogenesis were further evaluated. The CCK-8 assay and live/dead staining were used to assess the proliferation of BMSCs following treatment with different hydrogels. The results showed that the GCH/VMBG hydrogel significantly promoted BMSC proliferation (*p* < 0.001), with a cell viability of 118.2% after 3 days of co-culturing (Figure 13).

**Figure 14:**
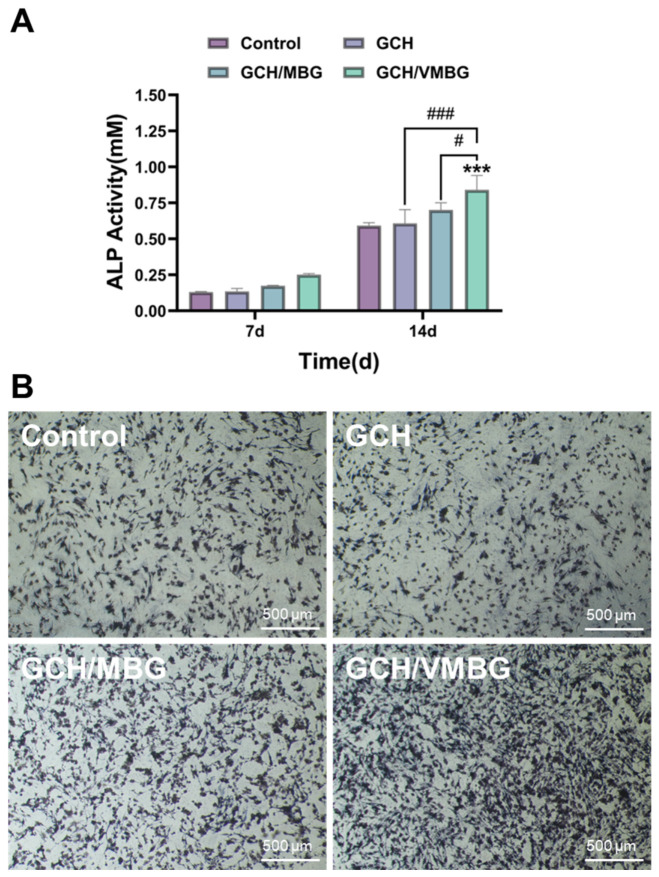
Effect of hydrogel on ALP activity of BMSC: (**A**) ALP activity and (**B**) ALP staining of BMSC cultured for 7 days in media containing different hydrogel extracts. (*** *p* < 0.001; # *p* < 0.05, ### *p* < 0.001).

Alkaline phosphatase (ALP) is a key early marker of osteogenic differentiation in BMSCs. Elevated ALP activity directly indicates osteoblast activation and initiation of bone matrix mineralization [38]. Quantitative analysis and staining of ALP were performed on BMSCs cultured in media supplemented with extracts from different hydrogels. The results demonstrated no significant difference in ALP expression between the GCH and control groups (*p* > 0.05), indicating limited osteogenic induction capacity of GCH. In contrast, the GCH/VMBG group showed significantly enhanced ALP activity (*p* < 0.001), likely attributable to the regulatory role of V(V) in promoting early-stage osteogenic differentiation (Figure 14).

**Figure 15:**
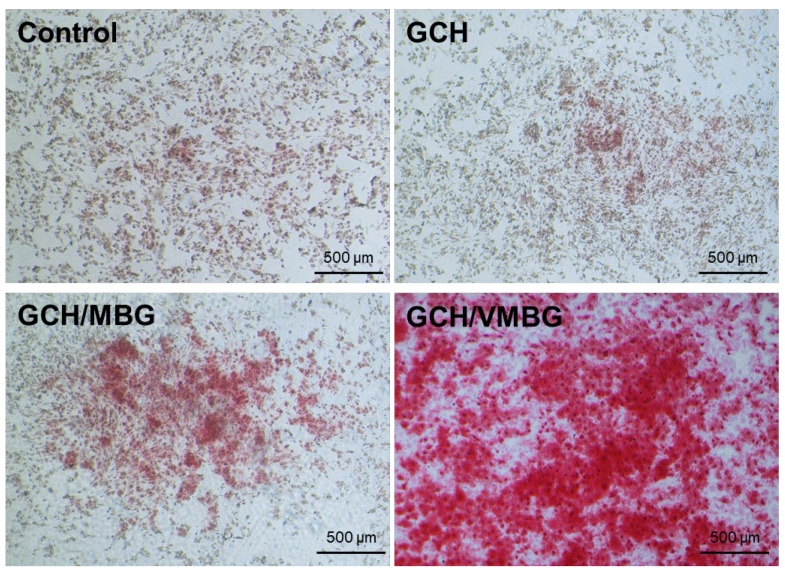
ARS staining of BMSC cultured in media supplemented with different hydrogel extracts for 14 days.

Calcium nodule formation is a distinct hallmark of mature bone tissue. To evaluate this process, ARS staining was conducted on BMSCs cultured in media supplemented with different hydrogel extracts. ARS staining revealed that minimal calcium nodules were formed in the control and GCH groups, whereas marked calcium nodule deposition was observed in the GCH/VMBG group (Figure 15), demonstrating that VMBG incorporation endowed hydrogels with an enhanced capacity to promote late-stage osteogenic maturation.

**Figure 16:**
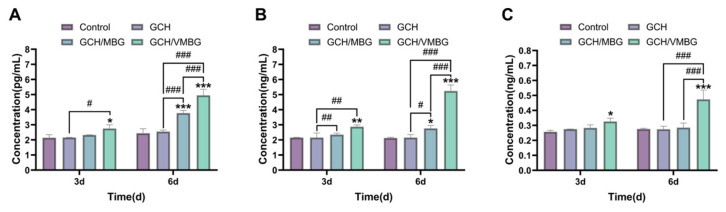
Effects of hydrogels on the expression of osteogenesis-related proteins: (**A**) BMP-2, (**B**) Col-I, and (**C**) OCN. (* *p* < 0.05, ** *p* < 0.01, *** *p* < 0.001; # *p* < 0.05, ## *p* < 0.01, ### *p* < 0.001).

BMP-2, Col-I, and OCN are key proteins involved in osteogenic proliferation and differentiation. We further analyzed the effects of the hydrogels on the expression of these proteins using ELISA. The results indicated that the GCH/VMBG group demonstrated significantly increased outcomes (*p* < 0.001) after 6 days of co-culturing (Figure 16), potentially attributable to the osteogenic-promoting effect of V species. During bone formation, BMSCs are recruited to the injury site and subsequently differentiated into osteoblasts, facilitating new bone formation [39]. As GCH/VMBG degrades, the released bioactive components significantly enhance the osteogenic differentiation of BMSCs. More importantly, the released V species promotes osteoblast proliferation and regulates osteogenic differentiation across early, middle, and late stages. In the early stage of osteogenic differentiation, V(V) activates the WNT/β-catenin and PI3K/ras/ERK signaling pathways by enhancing nuclear translocation efficiency, thereby promoting BMSC proliferation and upregulating BMP-2 expression. In the intermediate stage, V(V/IV) promotes the expression of ALP and Col-I through activation of the PI3K-MEK-ERK signaling pathway. In the late stage, V(IV) facilitates OCN expression and accelerates calcium nodule formation via activation of the Itga2b-FAK-MAPK pathway. Additionally, the MBG degradation is accompanied by the HAP formation on its surface, and HAP is closely associated with collagen fibers produced by osteoblasts. Collectively, the findings from the GCH/VMBG study demonstrated that this hydrogel promoted BMSC proliferation and differentiation by sustainedly releasing V species and other bioactive ions, thereby accelerating bone regeneration.

## 4. Conclusions

In this study, we developed a photo-curable injectable double-network hydrogel (GCH/VMBG) to address clinical challenges such as tumor-associated bone defects. Our findings demonstrate that GCH/VMBG can fill irregular bone cavities and rapidly form a stable matrix upon injection. The incorporation of VMBG can inhibit tumor recurrence associated with residual tumor cells and promote the osteogenic differentiation of BMSCs. After three days of co-culture with GCH/VMBG, the cell viabilities of UMR-106 and BMSC were 73.6% and 118.2%, respectively. Collectively, this work demonstrates that the injectable double-network hydrogel exhibits significant promise for bone defect treatment after osteosarcoma resection, combining antitumor efficacy with the capacity to promote osteogenesis.

## Data Availability

The original contributions presented in this study are included in the article. Further inquiries can be directed to the corresponding author.

## Abbreviations

The following abbreviations are used in this manuscript:

MBG	Mesoporous bioactive glass
VMBG	Vanadium-doped mesoporous bioactive glass
MA	Methacryloyl
GelMA	Gelatin methacryloyl
CMCS	Carboxymethyl chitosan
HA	Hyaluronic acid
CTAB	Cetyltrimethylammonium bromide
TEP	Triethyl phosphate
ASA	Ascorbic acid
TEOS	Tetraethyl orthosilicate
EA	Ethyl acetate
PBS	Phosphate-buffered saline
SBP	Simulated body fluid
UPW	Ultrapure water
CCK-8	Cell counting Kit-8
ALP	Alkaline phosphatase
ARS	Alizarin red S

## References

[1] Beird H.C., Bielack S.S., Flanagan A.M., Gill J., Heymann D., Janeway K.A., Livingston J.A., Roberts R.D., Strauss S.J., Gorlick R. (2022). Osteosarcoma. Nat. Rev. Dis. Primers.

[2] Cole S., Gianferante D.M., Zhu B., Mirabello L. (2022). Osteosarcoma: A Surveillance, Epidemiology, and End Results Program-Based Analysis from 1975 to 2017. Cancer.

[3] He M., Zhu C., Xu H., Sun D., Chen C., Feng G., Liu L., Li Y., Zhang L. (2020). Conducting Polyetheretherketone Nanocomposites with an Electrophoretically Deposited Bioactive Coating for Bone Tissue Regeneration and Multimodal Therapeutic Applications. ACS Appl. Mater. Interfaces..

[4] Kaseb H., Tan C., Townsend J.P., Costa J., Laskin W.B. (2024). Genomic Landscape of Osteosarcoma of Bone in an Older-Aged Patient Population and Analysis of Possible Etiologies Based on Molecular Signature. Genet. Test. Mol. Biomark..

[5] Guo C., Liao K., Xu K., Zhang J., Chen G., Luo X., Yang J., Dai Z., Lv X., Zhang F. (2026). DLST Mediates the Malignant Progression of Osteosarcoma Cells by Regulating the P38 MAPK Signaling Pathway. Biochem. Biophys. Res. Commun..

[6] Zhu C., He M., Sun D., Huang Y., Huang L., Du M., Wang J., Wang J., Li Z., Hu B. (2021). 3D-Printed Multifunctional Polyetheretherketone Bone Scaffold for Multimodal Treatment of Osteosarcoma and Osteomyelitis. ACS Appl. Mater. Interfaces.

[7] He M., Zhu C., Sun D., Liu Z., Du M., Huang Y., Huang L., Wang J., Liu L., Li Y. (2022). Layer-by-Layer Assembled Black Phosphorus/Chitosan Composite Coating for Multi-Functional PEEK Bone Scaffold. Compos. Part B Eng..

[8] Yuan Y., Zhang Q., Lin S., Li J. (2025). Water: The Soul of Hydrogels. Prog. Mater. Sci..

[9] Dinh L., Hwang S., Yan B. (2025). Hydrogel Conjugation: Engineering of Hydrogels for Drug Delivery. Pharmaceutics.

[10] Huang S., Hong X., Zhao M., Liu N., Liu H., Zhao J., Shao L., Xue W., Zhang H., Zhu P. (2022). Nanocomposite Hydrogels for Biomedical Applications. Bioeng. Transl. Med..

[11] Dong S., An S., Saiding Q., Chen Q., Liu B., Kong N., Chen W., Tao W. (2025). Therapeutic Hydrogels: Properties and Biomedical Applications. Chem. Rev..

[12] Farrukh A., Nayab S. (2024). Shape Memory Hydrogels for Biomedical Applications. Gels.

[13] Zhu J., Chen X., Chen Y., Huang C., Zhong N., Hu Y. (2024). Preparation and Characterization of Ternary Polysaccharide Hydrogels Based on Carboxymethyl Cellulose, Carboxymethyl Chitosan, and Carboxymethyl Β-Cyclodextrin. Int. J. Biol. Macromol..

[14] Kruczkowska W., Klosinski K.K., Grabowska K.H., Galeziewska J., Gromek P., Kciuk M., Kaluzinska-Kolat Z., Kolat D., Wach R.A. (2024). Medical Applications and Cellular Mechanisms of Action of Carboxymethyl Chitosan Hydrogels. Molecules.

[15] Zheng Z., Patel M., Patel R. (2022). Hyaluronic Acid-Based Materials for Bone Regeneration: A Review. React. Funct. Polym..

[16] Cui X., Huang C., Chen Z., Zhang M., Liu C., Su K., Wang J., Li L., Wang R., Li B. (2021). Hyaluronic Acid Facilitates Bone Repair Effects of Calcium Phosphate Cement by Accelerating Osteogenic Expression. Bioact. Mater..

[17] Bertsch P., Diba M., Mooney D.J., Leeuwenburgh S.C.G. (2022). Self-Healing Injectable Hydrogels for Tissue Regeneration. Chem. Rev..

[18] Zhang Y., Chen H., Li J. (2022). Recent Advances On Gelatin Methacrylate Hydrogels with Controlled Microstructures for Tissue Engineering. Int. J. Biol. Macromol..

[19] Choudhury S., Joshi A., Dasgupta D., Ghosh A., Asthana S., Chatterjee K. (2024). 4D printed biocompatible magnetic nanocomposites toward deployable constructs. Mater. Adv..

[20] Salama A.M., Hardy J.G., Yessuf A.M., Chen J., Ni M., Huang C., Zhang Q., Liu Y. (2025). Injectable Hydrogel Technologies for Bone Disease Treatment. ACS Appl. Bio Mater..

[21] Choudhury S., Joshi A., Agrawal A., Nain A., Bagde A., Patel A., Syed Z.Q., Asthana S., Chatterjee K. (2024). NIR-Responsive Deployable and Self-Fitting 4D-Printed Bone Tissue Scaffold. ACS Appl. Mater. Interfaces.

[22] Van Den Bulcke A.I., Bogdanov B., De Rooze N., Schacht E.H., Cornelissen M., Berghmans H. (2000). Structural and Rheological Properties of Methacrylamide Modified Gelatin Hydrogels. Biomacromolecules.

[23] Wang Q., Xu W., Koppolu R., van Bochove B., Seppälä J., Hupa L., Willför S., Xu C., Wang X. (2022). Injectable Thiol-Ene Hydrogel of Galactoglucomannan and Cellulose Nanocrystals in Delivery of Therapeutic Inorganic Ions with Embedded Bioactive Glass Nanoparticles. Carbohydr. Polym..

[24] Song X., Yu S., Deng J., Liu J., Xing J. (2024). An Organic–Inorganic Hybrid Hydrogel Based on Chitosan for Effective Hemostasis. ACS Appl. Polym. Mater..

[25] Vallet-Regí M., Colilla M., Izquierdo-Barba I., Vitale-Brovarone C., Fiorilli S. (2022). Achievements in Mesoporous Bioactive Glasses for Biomedical Applications. Pharmaceutics.

[26] Xiao J., Wei Q., Xue J., Yang Z., Deng Z., Zhao F. (2022). Preparation and in Vitro Bioactivity Study of a Novel Hollow Mesoporous Bioactive Glass Nanofiber Scaffold. Molecules.

[27] Panchal S.K., Wanyonyi S., Brown L. (2017). Selenium, Vanadium, and Chromium as Micronutrients to Improve Metabolic Syndrome. Curr. Hypertens. Rep..

[28] Treviño S., Díaz A., Sánchez-Lara E., Sanchez-Gaytan B.L., Perez-Aguilar J.M., González-Vergara E. (2018). Vanadium in Biological Action: Chemical, Pharmacological Aspects, and Metabolic Implications in Diabetes Mellitus. Biol. Trace Elem. Res..

[29] Liu X., Zhang P., Xu M., Zhao Z., Yin X., Pu X., Wang J., Liao X., Huang Z., Cao S. (2025). Mixed-Valence Vanadium-Doped Mesoporous Bioactive Glass for Treatment of Tumor-Associated Bone Defects. J. Mater. Chem. B.

[30] Cong D., Zhang Z., Xu M., Wang J., Pu X., Huang Z., Liao X., Yin G. (2023). Vanadium-Doped Mesoporous Bioactive Glass Promotes Osteogenic Differentiation of RBMSCs Via the WNT/β-Catenin Signaling Pathway. ACS Appl. Bio Mater..

[31] Megha M., Mohan C.C., Joy A., Unnikrishnan G., Thomas J., Haris M., Bhatt S.G., Kolanthai E., Senthilkumar M. (2024). Vanadium and Strontium Co-Doped Hydroxyapatite Enriched Polycaprolactone Matrices for Effective Bone Tissue Engineering: A Synergistic Approach. Int. J. Pharm..

[32] Liang Q., Hu Q., Miao G., Yuan B., Chen X. (2015). A Facile Synthesis of Novel Mesoporous Bioactive Glass Nanoparticles with Various Morphologies and Tunable Mesostructure by Sacrificial Liquid Template Method. Mater. Lett..

[33] Paek K., Woo S., Song S.J., Kim M.K., Yi K., Chung S., Kim J.A. (2024). A Well Plate-Based GelMA Photo-Crosslinking System with Tunable Hydrogel Mechanical Properties to Regulate the PTH-Mediated Osteogenic Fate. Biofabrication.

[34] Armiento A.R., Hatt L.P., Rosenberg G.S., Thompson K., Stoddart M.J. (2020). Functional Biomaterials for Bone Regeneration: A Lesson in Complex Biology. Adv. Funct. Mater..

[35] Jian G., Li D., Ying Q., Chen X., Zhai Q., Wang S., Mei L., Cannon R.D., Ji P., Liu W. (2023). Dual Photo-Enhanced Interpenetrating Network Hydrogel with Biophysical and Biochemical Signals for Infected Bone Defect Healing. Adv. Healthc. Mater..

[36] He M., Yang X., Xiang D., Chan Y.K., Yin G., Yang W., Deng Y. (2025). Jahn–Teller-Driven Electronic Modulation of Bio-Heterojunction for Wound Regeneration after Postoperative Tumor Resection. Nano Lett..

[37] Wang Q., Zang C., Chan Y.K., Wang S., Yang W., Deng Y., Zhu T., He M. (2023). CoFe_2_O_4_/MXene nanosheets modified hydrogel on PEEK with phototherapeutic and GPx-Mimetic activities for anti-pathogens in infectious bone defect repairment. Mater. Chem. Phys..

[38] Zhu C., He M., Wang J., Huang Y., Deng W., Liu L., Feng G., Zhang L., Song Y. (2024). Bioactivated Polyetheretherketone Scaffold Able to Generate Mild Heat for Promoting Bone Regeneration via Activating MAPK/ERK Signaling Pathway. J. Mater. Sci. Technol..

[39] He M., Wang Q., Lin Z., Sun D., Yin G., Deng Y., Yang W. (2025). Push–Pull Electronic Effect and D-Band Center Bi-Modulated Bio-Heterojunction Enzyme Enables All-Stage Infected Wound Healing. Adv. Mater..

